# Selection of Superior Yeast Strains for the Fermentation of Lignocellulosic Steam-Exploded Residues

**DOI:** 10.3389/fmicb.2021.756032

**Published:** 2021-11-04

**Authors:** Lorenzo Cagnin, Nicoletta Gronchi, Marina Basaglia, Lorenzo Favaro, Sergio Casella

**Affiliations:** Department of Agronomy, Food, Natural Resources, Animals and Environment (DAFNAE), University of Padova, Legnaro, Italy

**Keywords:** bioethanol, sugarcane bagasse, cardoon, common reed, industrial yeast strains, steam explosion

## Abstract

The production of lignocellulosic ethanol calls for a robust fermentative yeast able to tolerate a wide range of toxic molecules that occur in the pre-treated lignocellulose. The concentration of inhibitors varies according to the composition of the lignocellulosic material and the harshness of the pre-treatment used. It follows that the versatility of the yeast should be considered when selecting a robust strain. This work aimed at the validation of seven natural *Saccharomyces cerevisiae* strains, previously selected for their industrial fitness, for their application in the production of lignocellulosic bioethanol. Their inhibitor resistance and fermentative performances were compared to those of the benchmark industrial yeast *S. cerevisiae* Ethanol Red, currently utilized in the second-generation ethanol plants. The yeast strains were characterized for their tolerance using a synthetic inhibitor mixture formulated with increasing concentrations of weak acids and furans, as well as steam-exploded lignocellulosic pre-hydrolysates, generally containing the same inhibitors. The eight non-diluted liquors have been adopted to assess yeast ability to withstand bioethanol industrial conditions. The most tolerant *S. cerevisiae* Fm17 strain, together with the reference Ethanol Red, was evaluated for fermentative performances in two pre-hydrolysates obtained from cardoon and common reed, chosen for their large inhibitor concentrations. *S. cerevisiae* Fm17 outperformed the industrial strain Ethanol Red, producing up to 18 and 39 g/L ethanol from cardoon and common reed, respectively, with ethanol yields always higher than those of the benchmark strain. This natural strain exhibits great potential to be used as superior yeast in the lignocellulosic ethanol plants.

## Introduction

Several cheap forestry and agricultural waste streams, as well as energy crops, are available for being applied as feedstocks for bioethanol production (Bhatia et al., 2017). However, such biomasses need to be pre-treated to make the cellulose more accessible to the following enzymatic hydrolysis aimed to release fermentable sugars.

Although demonstration plants using sugarcane bagasse, corn stover, wheat straw, and switchgrass are now in operation (Bhatia et al., 2017; Jansen et al., 2017), before reaching the final large-scale application of lignocellulosic ethanol, several challenges must be faced (Chandel et al., 2018; Dale, 2018; Liu et al., 2019) mainly about both the pre-treatment technologies and the yeast used in the processes.

Many pre-treatment technologies have been developed in the last decades and have important effects on downstream procedures, yields, and costs (da Costa Sousa et al., 2009; Nair et al., 2017; Awasthi et al., 2020; Park et al., 2020). Among the pre-treatments, steam explosion unsettles lignocellulosic materials by physical and chemical reactions, allowing a more effective subsequent enzymatic digestion. However, during steam explosion, possible inhibitors of fermentations such as phenolic compounds, furans, or weak acids are released decreasing the final ethanol yields (García et al., 2014; Morales et al., 2017).

Moreover, the fermenting yeast strains used in the second-generation ethanol plants, including *S. cerevisiae* Ethanol Red, have been originally selected for the application in first-generation ethanol distilleries. As such, there strains are generally unsuitable for the harsher conditions typical of lignocellulosic ethanol (Jönsson et al., 2013; Jansen et al., 2017; Chandel et al., 2018; Favaro et al., 2019b).

Unfortunately, while several research projects focused on the search for efficient pre-treatment technologies to maximize sugar yield (reviewed in Galbe and Zacchi, 2007; Bhutto et al., 2017; Liu et al., 2020), only a limited number approached yeast strains selection on the basis of their fermentative performances, innate resistance, and industrial fitness (Basso et al., 2008; Albers and Larsson, 2009; Pereira et al., 2011, 2014; Favaro et al., 2013a; Dubey et al., 2016; Kim et al., 2017; de Witt et al., 2019). This is a big gap of knowledge in order to optimize both substrate conversion and energy efficiency of lignocellulosic ethanol (Chandel et al., 2018).

First-generation ethanol experiences demonstrated that the efficient conversion of the raw material (corn or sugarcane) into alcohol is crucial for process economy: bioethanol industry should aim for at least 90% of theoretical yields (Walker and Walker, 2018) and even an increase of 1% would result in a considerable increase of the profit (Della-Bianca et al., 2013; Dmytruk et al., 2017). The same concept has still to be transferred to ethanol production from lignocellulose where the ethanol yields are below the industrial thresholds (Walker and Walker, 2018; Favaro et al., 2019b). Thus, the search of a vigorous yeast strain able to efficiently ferment in such industrial conditions is essential in a lignocellulosic ethanol context.

Conventional screenings for naturally tolerant *S. cerevisiae* strains were usually directed to individual stressors (Garay-Arroyo et al., 2004; Abdel-Banat et al., 2010; Mertens et al., 2018; Cunha et al., 2019). However, discovering and selecting strains with tolerance to multiple stresses, as well as assessing their fitness in simulated industrial conditions (co-presence of inhibitors, pH decrease, high osmolarity), would be a more realistic approach toward the development of the second-generation bioethanol industry as well reported in recent literature (Mertens et al., 2018; Brandt et al., 2019; Cunha et al., 2019; de Witt et al., 2019; Huang et al., 2019; Park et al., 2020; van Dijk et al., 2020).

In the present research paper, sugarcane bagasse, common reed, and cardoon were considered due to their potential as a source of renewable energy and their sustainability in fermentation to fuel route (Cotana et al., 2015a,b; Espada et al., 2021). Eight undiluted inhibitor-rich liquors, obtained after the steam explosion of the feedstocks mentioned above, were here used as such for both strain inhibitor tolerance assessment and fermentation to ethanol. The industrial fitness of seven selected *S. cerevisiae* strains, previously described for their high thermo- and inhibitor-tolerance (Favaro et al., 2013a, 2014; Jansen et al., 2018), was evaluated at lab scale and compared to that of the industrial reference *S. cerevisiae* Ethanol Red, one of the most used strain in the lignocellulose-to-ethanol processes (Dmytruk et al., 2017; Walker and Walker, 2018). The use of undetoxified steam-exploded liquors was useful to simulate the industrial environment as closely as possible.

The natural yeast strain showing the most promising inhibitor tolerance in many of the screened pre-hydrolysates, together with the reference *S. cerevisiae* Ethanol Red, were further adopted for the fermentation of two pre-hydrolysates, chosen for their high inhibitor concentration. These liquors, deriving from cardoon and common reed, were also supplemented up to 40 and 92 g/L of glucose, respectively, to simulate the highest glucose concentration obtained by enzymatic saccharification of each steam-exploded water insoluble solid (WIS; Cotana et al., 2015a,b; Cavalaglio et al., 2016).

## Materials and Methods

### Feedstocks and Chemicals

In order to obtain steam-exploded liquors with high inhibitor concentrations, samples of *Phragmites australis* (common reed), *Cynara cardunculus* (cardoon), and *Saccharum officinarum* (sugarcane) bagasse pre-treated by applying different conditions (residence time and temperature) resulting in increasing severity factors (Log*R*_0_) were investigated in this study (Table 1). Log*R*_0_-values (Overend and Chornet, 1987; Espirito Santo et al., 2020) were obtained according to Equation [1]:

**TABLE 1:** Pre-treatment parameters, pH, and composition of the pre-hydrolysates used in this study.

				**g/L**							
**Substrates**	**Name**	**Log*R*_0_**	**pH**	**Glucose**	**Arabinose**	**Xylose**	**Formic acid**	**Acetic acid**	**Levulinic acid**	**Furfural**	**HMF**
*P. australis*	Pa1	3.60	3.75	0.14 ± 0.01	0.25 ± 0.02	1.24 ± 0.11	0.32 ± 0.01	1.00 ± 0.08	n.d.	0.24 ± 0.01	0.05 ± 0.01
	Pa2	4.00	3.29	0.29 ± 0.02	0.35 ± 0.01	2.04 ± 0.18	0.78 ± 0.05	2.18 ± 0.11	0.001 ± 0.001	0.97 ± 0.07	0.13 ± 0.01
	Pa3	4.40	3.23	0.43 ± 0.02	0.09 ± 0.01	0.53 ± 0.04	1.28 ± 0.11	3.50 ± 0.25	0.008 ± 0.001	1.43 ± 0.11	0.48 ± 0.03
*C. cardunculus*	Cc1	3.85	4.10	0.02 ± 0.01	0.01 ± 0.01	0.23 ± 0.01	0.50 ± 0.03	0.71 ± 0.06	0.002 ± 0.001	0.09 ± 0.01	0.05 ± 0.01
	Cc2	4.02	3.96	0.30 ± 0.02	0.11 ± 0.02	2.15 ± 0.12	1.73 ± 0.11	2.15 ± 0.11	0.003 ± 0.001	0.36 ± 0.02	0.20 ± 0.02
	Cc3	4.28	3.83	0.20 ± 0.02	0.20 ± 0.02	2.20 ± 0.17	2.18 ± 0.15	2.76 ± 0.24	0.004 ± 0.001	0.44 ± 0.02	0.28 ± 0.02
	Cc4	4.53	3.49	0.13 ± 0.01	0.03 ± 0.01	1.91 ± 0.11	4.28 ± 0.28	5.80 ± 0.41	0.011 ± 0.003	0.64 ± 0.04	0.39 ± 0.02
*S. officinarum*	So1	4.65	3.28	0.50 ± 0.03	0.40 ± 0.03	2.95 ± 0.18	3.00 ± 0.19	11.20 ± 0.90	0.019 ± 0.005	1.70 ± 0.12	0.50 ± 0.03

*Severity factor Log*R*_0_ correlates with the harshness of the pre-treatment (Cotana et al., 2015a, b). n.d., not detected.*


(1) $$R_{0}=t\,e^{\left[\left(T-100\right)\,/\,14.75\right]}$$

where t is the residence time (sec/min) and T is the temperature (°C).

Briefly, pre-treatment liquors of cardoon and common reed were obtained by steam explosion. For every Log*R*_0_-value, six consecutive explosions were executed using 500 g of dry biomass for each explosion. Liquors were then separated from the WIS fraction using a stainless-steel filter with a cutoff of 1 mm (Cotana et al., 2015a,b; Cagnin et al., 2018).

Sugarcane pre-hydrolysate was obtained in a steam explosion plant composed of a 19 L reactor, a collection tank, and a 40-bar electrical boiler. Milled sugarcane bagasse samples, dried in a drying chamber to a final moisture content of 10% (w/w), were loaded into the reactor and treated for 10 min at 200°C. The pre-hydrolysate was then removed using a locally manufactured dead-end press. All the pre-hydrolysates were refrigerated until use. The inhibitor and sugar contents are reported in Table 1.

All chemicals, media components, and supplements were of analytical grade standard.

### Yeast Strains

The phenotypes and sources of the *S. cerevisiae* strains used in this work are summarized in Table 2. Yeast strains pre-cultures were grown in YPD medium (g/L: yeast extract, 10; peptone, 20; glucose, 20) at 30°C on a rotary shaker set at 130 rpm unless otherwise stated.

**TABLE 2:** Yeast strains used in this study.

**Strain**	**Relevant phenotype**	**References**	**Source**
*S. cerevisiae* Ethanol Red	Industrial strain for bioethanol production	Lesaffre (Marcq-en-Barśul, France)	Fermentis division
*S. cerevisiae* Fm17	Newly isolated strain with high inhibitor tolerance	Favaro et al., 2013a	DAFNAE collection (University of Padova, Italy)
*S. cerevisiae* Fm89	Newly isolated strain with high inhibitor tolerance	Favaro et al., 2013a	DAFNAE collection (University of Padova, Italy)
*S. cerevisiae* Fm90	Newly isolated strain with high inhibitor tolerance	Favaro et al., 2013a	DAFNAE collection (University of Padova, Italy)
*S. cerevisiae* Fm96	Newly isolated strain with high inhibitor tolerance	Favaro et al., 2014	DAFNAE collection (University of Padova, Italy)
*S. cerevisiae* M2n	Industrial distillery strain	Viktor et al., 2013	Stellenbosch University (South Africa)
*S. cerevisiae* MEL2	Industrial strain with high fermentative vigor	Favaro et al., 2013b	DAFNAE collection(University of Padova, Italy)
*S. cerevisiae* YI30	Wild type strain with high inhibitor tolerance	Jansen et al., 2018	Stellenbosch University (South Africa)

### Evaluation of Inhibitor Tolerance of Selected Wild Type and Industrial Yeast

Seven natural yeast strains (Fm17, Fm89, Fm90, Fm96, M2n, MEL2, and YI30) were screened for their industrial fitness using Ethanol Red as benchmark industrial yeast. Inhibitor tolerance in the presence of four synthetic inhibitor mixtures and eight inhibitor-rich lignocellulosic pre-hydrolysates was assessed.

#### Inhibitor Tolerance in Synthetic Inhibitor Mixtures

Yeast strains were firstly evaluated for their inhibitor tolerance in filter-sterilized (0.22 μm) defined Yeast Nitrogen Base (YNB) medium supplemented with 20 g/L of glucose and containing increasing concentrations of weak acids (acetic, formic acids) and furans (furfural, HMF: 5-hydroxymethyl-2-furaldehyde). Inhibitors were formulated into four mixtures, namely, RC_25_, RC_50_, RC_100_, and RC_200_ (RC: Relative Concentration) obtained by adding increasing doses of each toxic compound. RC_100_ was formulated using the highest concentrations of each tested inhibitor present in many lignocellulosic pre-hydrolysates, namely, acetic acid 7.20, formic acid 2.40, furfural 2.70, HMF 3.78 g/L (Favaro et al., 2016, 2019a; Jönsson and Martín, 2016; Bhatia et al., 2017; Bhutto et al., 2017; Dmytruk et al., 2017; Jansen et al., 2018; Roscini et al., 2019). RC_25_ and RC_50_ mixtures were, respectively, obtained as 4-fold and 2-fold dilutions of RC_100_. RC_200_ is a 2-fold concentration of RC_100_. pH was adjusted to 5.0, using 5 M NaOH. This particular pH is widely used in the bioethanol production process (Kádár et al., 2007). The detailed inhibitor composition of each mixture is reported in Table 3.

**TABLE 3:** Inhibitor composition of four quaternary mixtures used to assess yeast inhibitor tolerance.

	**Concentration (g/L)**			
**Inhibitor**	**RC_25_**	**RC_50_**	**RC_100_**	**RC_200_**
Acetic acid	1.80	3.60	7.20	14.40
Formic acid	0.60	1.20	2.40	4.80
Furfural	0.68	1.35	2.70	5.40
HMF	0.95	1.89	3.78	7.56

*Before adjustment, pH-values of inhibitor mixtures RC_25_, RC_50_, RC_100_, and RC_200_ were 2.60, 2.50, 2.40, and 2.20, respectively.*

Overnight cultures of each yeast strain, grown at 30°C in YNB medium containing 20 g/L of glucose, were transferred, in biological triplicate, at an inoculum concentration of 1 × 10^6^ cells/mL, in 2 mL Eppendorf tubes containing 0.9 mL of medium. After 40 h of growth (30°C, 130 rpm), the optical density at 600 nm (OD_600_) was measured. For each strain, the tolerance was estimated as relative growth (%), calculated as the ratio between measured OD_600_-values of the medium with inhibitors and the control medium, devoid of any inhibitor mixture (Favaro et al., 2014).

#### Inhibitor Tolerance in Lignocellulosic Pre-hydrolysates

Inhibitor tolerance of the strains was also assayed in eight lignocellulosic pre-hydrolysates, obtained by steam explosion of *P. australis*, *C. cardunculus*, and *S. officinarum* bagasse (Table 1).

Overnight cultures of each strain were used to inoculate a volume of 200 μl of each lignocellulosic hydrolysate containing YNB and 20 g/L of glucose. pH of the medium was not modified. The medium was filter-sterilized through 0.22 μm. The experiment was carried out in quintuplicate for each condition in 96-well plates using the multimode microplate reader TECAN Spark 10 M (Tecan Group Ltd., Switzerland). An increase in OD_600_-value indicated the ability of the strain to sustain growth in the presence of the specific pre-hydrolysate.

Similarly, yeast strains were evaluated in 0.9 mL of YNB medium formulated with pre-hydrolysates Pa3, Cc3, Cc4, and containing 20 g/L glucose. pH was either not modified or adjusted at values of 4.5 and 5.0 by adding 5 M NaOH. The experiment was carried out in triplicate for each condition. Cell culture preparation, analytical methods, and evaluation of inhibitor tolerance in terms of relative growth were performed as defined in the section “Inhibitor Tolerance in Synthetic Inhibitor Mixtures.”

### Fermentation of Lignocellulosic Pre-hydrolysates

Fermentation performances of *S. cerevisiae* Fm17 and Ethanol Red were evaluated in YNB medium formulated with cardoon (Cc3) or common reed (Pa3) pre-hydrolysates supplemented with up to 20 g/L of glucose. Moreover, the two pre-treatment liquors were supplemented with YNB and 40 or 92 g/L of glucose, respectively, to simulate the highest glucose concentration obtained by enzymatic saccharification of each steam-exploded WIS (Cotana et al., 2015a, b; Cavalaglio et al., 2016). After pH was adjusted to 5.0 using 5 M NaOH, broths were sterilized using a 0.22 μm sterile filter.

Pre-cultures of yeast cells grown to stationary phase in YNB medium containing 20 g/L of glucose were used to inoculate 50 mL medium to an initial OD_600_ of 1.0 in 55 mL glass serum bottles. The small-scale fermentations were carried out in triplicate under oxygen-limited conditions. Bottles were sealed with rubber stoppers, incubated at 30°C, and mixed on a magnetic stirrer. Growth was measured as OD_600_ and samples, taken through a capped syringe needle pierced through the bottle stopper, were stored at −20°C. Collected samples were filtered through a 0.22 μm pore filter and diluted prior to HPLC (high-performance liquid chromatography) analysis performed as described in the section “Analytical Methods, Calculations, and Statistical Analysis.”

### Analytical Methods, Calculations, and Statistical Analysis

Samples of lignocellulosic pre-hydrolysates and liquid fractions during small scale fermentations were analyzed for ethanol, glycerol, arabinose, galactose, glucose, xylose, mannose, sucrose, maltose, cellobiose, acetic acid, formic acid, levulinic acid, furfural, and HMF. Liquid chromatography analysis was performed using a Shimadzu Nexera HPLC system, equipped with a RID-10A refractive index detector (Shimadzu, Kyoto, Japan). The chromatographic separations were performed using a Phenomenex Rezex ROA-Organic Acid H^+^ (8%) column (300 mm × 7.8 mm). The column temperature was set at 65°C, and the analysis was performed at a flow rate of 0.6 mL/min using isocratic elution, with 0.01 M H_2_SO_4_ as a mobile phase (Favaro et al., 2010). Analytes were identified by comparing their retention times, and the concentrations were calculated using calibration curves of the corresponding external standard.

The ethanol yield (*Y*_E/G_) from glucose was calculated as the highest amount of ethanol produced per gram of consumed glucose (g/g). The volumetric productivity (*Q*) was based on grams of the highest ethanol produced per liter of culture medium per hour (g/L h^–1^). *Q*_max_ was calculated as the highest volumetric productivity along the fermentations.

Statistical analyses were obtained using the Graphpad Prism 5 package (Graphpad Software, Inc., San Diego, CA, United States). Mean values, standard deviations, and descriptive statistics were calculated. Fermentations performances were analyzed by ANOVA (ANalysis Of Variance) using Duncan test *post hoc* means differentiation.

## Results and Discussion

### Screening of *Saccharomyces cerevisiae* Yeast Strains for Inhibitor Tolerance

Seven *S. cerevisiae* strains, namely, Fm17, Fm89, Fm90, Fm96, M2n, MEL2, and YI30, were previously described for their potential in various bioethanol applications (Favaro et al., 2013a, 2014; Jansen et al., 2018). In this study, these strains were further characterized with the final aim of assessing their promise to be used for lignocellulosic ethanol production. As such, *S. cerevisiae* Ethanol Red was specifically chosen as reference industrial yeast (Walker and Walker, 2018; Favaro et al., 2019b).

#### Inhibitor Tolerance in Synthetic Inhibitor Mixtures

Inhibitor resistance was firstly evaluated in the presence of four synthetic mixtures of inhibitors most commonly found in lignocellulosic pre-hydrolysates. *S. cerevisiae* strains were grown in YNB medium containing 20 g/L of glucose and increasing concentrations of synthetic inhibitors, weak acids (acetic, formic acid), and furans (furfural, HMF). As described in the section “Inhibitor Tolerance in Synthetic Inhibitor Mixtures,” the tolerance of each strain was evaluated as relative growth (%) by comparing the cell growth in the medium containing inhibitors with that lacking these compounds, after 40 h incubation at 30°C (Table 4).

**TABLE 4:** Influence of increasing concentrations of mixtures of weak acids (acetic and formic acid) and furans (furfural and HMF) on aerobic yeast growth in defined YNB medium supplemented with 20 g/L of glucose.

	**Relative growth (%)**							
	**Fm17**	**Fm89**	**Fm90**	**Fm96**	**M2n**	**MEL2**	**YI30**	**Ethanol Red**
RC_25_	94 ± 4	81 ± 4	87 ± 4	79 ± 4	50 ± 4	82 ± 4	85 ± 3	65 ± 3
RC_50_	71 ± 4	62 ± 3	59 ± 3	53 ± 3	21 ± 1	60 ± 3	63 ± 3	44 ± 2
RC_100_	60 ± 3	45 ± 2	42 ± 2	39 ± 3	14 ± 1	28 ± 1	55 ± 2	11 ± 1
RC_200_	0	0	0	0	0	0	0	0

*pH was adjusted to 5.0 with 5 M NaOH. Inhibitor tolerance values are expressed as relative growth (%) for each strain after 40 h. Results are the means of three replicates (± SD).*

Inhibitor mixtures hindered cell growth with different degrees of severity. As expected, the relative growth decreased by increasing the concentration of inhibitors. *S. cerevisiae* M2n and Ethanol Red displayed the lowest tolerance already in the presence of the most diluted mixture (RC_25_), with a relative growth of 50 and 65%, respectively. Conversely, Fm17 exhibited the highest degree of tolerance to the inhibitors formulations, with values of 94, 71, and 60% in RC_25_, RC_50_, and RC_100_, respectively. A slightly lower extent of tolerance has been also measured for *S. cerevisiae* YI30, recently proposed as a promising strain for lignocellulosic ethanol (Jansen et al., 2018). By contrast, RC_200_ inhibited growth of all strains tested.

#### Using Lignocellulosic Pre-hydrolysates to Assess Yeast Inhibitor Tolerance

Although the synthetic mixtures were often used for assessing the inhibitor tolerance of *S. cerevisiae* strains (Martín and Jönsson, 2003; Favaro et al., 2013a, 2014; Viktor et al., 2013; Wimalasena et al., 2014; Jansen et al., 2018), the ability of yeast cells to grow and withstand real lignocellulosic pre-treated materials could greatly vary, due to the hindering action of other toxic compounds that cannot be easily identified or quantified (Chandel et al., 2018). The objective of this work was to select yeast strains for second-generation bioethanol production in the industrial context, based on their high level of robustness and strong fermentative performances. Therefore, for the first time, several pre-hydrolysates from steam pre-treated lignocellulosic materials, namely, sugarcane bagasse, common reed, and cardoon, were used as a source of inhibitors. These feedstocks, selected as model of other cheap and abundant lignocellulosic substrates, together with steam explosion, which is one of the most commonly used pre-treatments (Mussatto, 2016), would result in conditions representative for second-generation ethanol production.

In order to obtain a cluster of liquors enriched in inhibitory compounds, several Log*R*_0_-values were applied for the steam-explosion of the lignocellulosic materials, resulting in the release of different inhibitor concentrations and small amounts of xylose, arabinose, and glucose (Table 1). The steam explosion of *S. officinarum* bagasse yielded the pre-hydrolysate So1, having the highest amounts of aliphatic acids (about 14.2 g/L) and furans (2.2 g/L). These values agree with those described in other steam-exploded sugarcane bagasse samples (Martìn et al., 2002; Fockink et al., 2018). The higher severity, the higher release of inhibitors in both *P. australis* and *C. cardunculus* pre-hydrolysates (Table 1). Among the *P. australis* pre-treatments liquors, Pa3 was the richest in terms of inhibitors with almost 4.8 and 2.0 g/L of weak acids and furans, respectively. On the other hand, Cc4 contained the uppermost levels of inhibitors among the pre-hydrolysates of *C. cardunculus*. Such concentrations compare well with those recently reported for cardoon and common read steam gun pre-treatments (Bułkowska and Klimiuk, 2016).

The ability of the yeast strains to grow in the presence of eight undiluted pre-hydrolysates was firstly evaluated in a qualitative high-throughput assay using YNB containing 20 g/L of glucose, as described in the section “Inhibitor Tolerance in Lignocellulosic Pre-hydrolysates.” Yeast growth was determined by detecting increased turbidity of the medium (Supplementary Table 1). All strains were able to grow in pre-hydrolysates Pa1 and Pa2 from *P. australis* and in Cc1, Cc2, and Cc3 from *C. cardunculus*, except for Fm89 strain in Pa2. Pre-treatment liquors Pa3 from *P. australis*, Cc4 from *C. cardunculus*, and So1 from *S. officinarum* bagasse did not support the growth of any yeast, indicating that their concentrations of toxic chemical species were higher than yeast could tolerate. This hypothesis is confirmed by the large inhibitor concentrations present in each of these pre-hydrolysates (Table 1). In fact, Pa3 contains the uppermost amount of inhibitors found in the pre-hydrolysates originating from *P. australis* and one of the strongest concentrations of furans among all the pre-hydrolysates. Similarly, for Cc4, which appears as the harshest liquor from *C. cardunculus*, with very high concentrations of weak acids (nearly 10 g/L).

With the aim to select highly tolerant yeast, Pa3, Cc3, and Cc4 liquors were chosen for additional experimental activities on yeast inhibitor resistance. Since the use of undiluted substrate would be the best criterion to adopt, So1 was excluded because of its high inhibitor content (Table 1) exceeding the ability of the yeast to cope with (Palmqvist and Hahn-Hägerdal, 2000; Jönsson et al., 2013; Jönsson and Martín, 2016). Relative inhibitor tolerance of the eight strains was quantified in YNB medium containing 20 g/L of glucose and formulated with pre-hydrolysates Pa3, Cc3, and Cc4, without altering the pH of the media. In these conditions, yeast growth was completely inhibited in Pa3 and Cc4, while all strains could grow in the presence of the pre-hydrolysate Cc3 (Table 5). *S. cerevisiae* YI30 and Fm17 exhibited the highest relative growth values, 70 and 62%, respectively. The reference Ethanol Red showed lower inhibitor tolerance. Higher toxicity of pre-hydrolysates Pa3 and Cc4 is likely caused by the higher amounts of acetic acid, furfural, and HMF (Table 1), compared to the less toxic Cc3.

**TABLE 5:** Influence of different lignocellulosic pre-hydrolysates on yeast growth in defined YNB medium supplemented with 20 g/L of glucose with or without pH adjustment to pH 5.0 with 5 M NaOH.

	**Pa3**		**Cc3**		**Cc4**	
**Strain**	**Unaltered**	**Adjusted**	**Unaltered**	**Adjusted**	**Unaltered**	**Adjusted**
	**(pH 3.23)**	**(pH 5.00)**	**(pH 3.83)**	**(pH 5.00)**	**(pH 3.49)**	**(pH 5.00)**
Fm17	0	69 ± 4	62 ± 3	88 ± 5	0	66 ± 4
Fm89	0	68 ± 4	48 ± 3	61 ± 3	0	63 ± 3
Fm90	0	61 ± 3	61 ± 4	80 ± 4	0	60 ± 4
Fm96	0	9 ± 1	50 ± 3	79 ± 4	0	54 ± 3
M2n	0	16 ± 1	53 ± 3	57 ± 3	0	60 ± 3
MEL2	0	8 ± 1	30 ± 2	61 ± 3	0	56 ± 3
YI30	0	57 ± 3	70 ± 4	81 ± 5	0	60 ± 3
Ethanol Red	0	37 ± 2	50 ± 3	78 ± 4	0	63 ± 3

*Inhibitor tolerance is expressed as relative growth (%) measured for each strain after 40 h in YNB, and results are the means of three replicates (± SD).*

Overall, the selected natural yeast strains exhibited a versatility toward multiple pre-treated materials greater than the benchmark industrial yeast (Supplementary Table 1 and Table 5). This is one of the main achievements for a lignocellulosic ethanol yeast (Dmytruk et al., 2017; Walker and Walker, 2018; Favaro et al., 2019b).

The experiment was replicated after adjusting medium acidity to pH 5.0 (Table 5). pH adjustment resulted in an overall improvement of relative growth: all strains did grow in the presence of pre-hydrolysates Pa3 and Cc3, as well as in Cc4. While all *S. cerevisiae* isolates showed similar tolerance to Cc4, amounting to about 60% relative growth (54–66%), strong differences could be identified in the case of the liquor Pa3 (8–69%) and Cc3 (57–88%).

The reference industrial yeast Ethanol Red proved to be extremely inhibited by Pa3 while showing high tolerance in Cc3 and Cc4. On the contrary, *S. cerevisiae* Fm17 exhibited the highest relative growth values once exposed to the three pre-hydrolysates at pH 5.0. Similar inhibitor tolerance patterns, although with lower values, were detected for *S. cerevisiae* Fm90 and YI30 (Table 5).

Benefits generated by pH adjustment can be ascribed to the acidity-related dissociation of weak acids. As extracellular undissociated acids are liposoluble, they can permeate through the cell membrane and lower the cytosolic pH, thus inducing stress levels to the cell that can cause the inhibition of metabolic activities. The amount of dissociated acid is a function of pH and the p*K*_*a*_ of each specific acid. The concentration of undissociated and dissociated acids in lignocellulosic pre-hydrolysates is then very sensitive to the medium acidity (Palmqvist and Hahn-Hägerdal, 2000; Landaeta et al., 2013; Jönsson and Martín, 2016). The increase of medium pH to values closer to, or higher than, the p*K*_*a*_ of weak acids reduces the concentration of harmful undissociated acids, resulting in less stressful conditions for the yeast. This is particularly true for the hydrolysate Cc4, quite rich in terms of formic and acetic acid (Table 1).

Based on the high inhibitor tolerance shown in different lignocellulosic pre-hydrolysates, *S. cerevisiae* Fm17 was selected and further characterized, together with the reference Ethanol Red, in terms of fermenting abilities in the lignocellulosic pre-treatment liquors of *P. australis* (Pa3) and *C. cardunculus* (Cc3).

### Fermentation Performances of Selected Yeast Strains in Lignocellulosic Pre-hydrolysates

**FIGURE 1:**
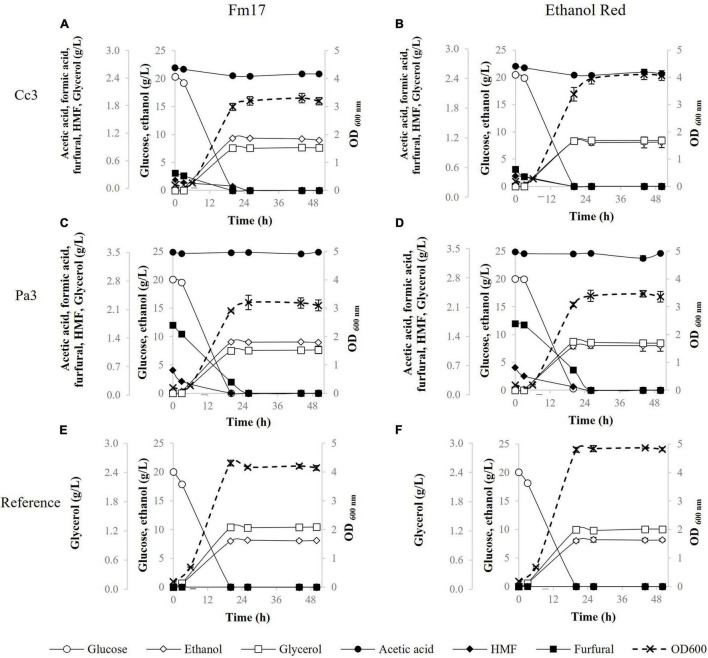
Fermentation performances in the presence of pre-hydrolysate Cc3 from *Cynara cardunculus* and Pa3 from *Phragmites australis*, supplemented with YNB containing 20 g/L of glucose, by *Saccharomyces cerevisiae* Fm17 **(A,C)** and Ethanol Red **(B,D)**. *S. cerevisiae* Fm17 **(E)** and Ethanol Red **(F)** were inoculated also in the reference broth (YNB containing 20 g/L of glucose without any pre-hydrolysate). When necessary, acidity of the medium was adjusted to pH 5.0 with NaOH. The experiment was conducted in triplicate. Error bars correspond to the standard deviation of the means.

Since developing industrial yeast with high fermentative capacity from different pre-treated feedstocks, rather than a preferred substrate, is one of the ultimate goals, pre-hydrolysates from cardoon (Cc3) and common reed (Pa3) were used as a substrate to simulate the industrial environment as closely as possible. Firstly, the pre-treatment liquors were supplemented up to 20 g/L glucose and used in small-scale fermentations to compare the fermenting abilities of *S. cerevisiae* Fm17 to those of the reference industrial strain Ethanol Red (Figures 1A–D and Table 6). The acidity of the medium was adjusted to pH 5.0 with 5 M NaOH. A fermentation medium formulated without Cc3 or Pa3 was used as control (Figures 1E,F and Table 6).

**TABLE 6:** Fermentative performances at 30°C of *Saccharomyces cerevisiae* strain Fm17 and the benchmark *S. cerevisiae* Ethanol Red (ER) when incubated in the presence of pre-hydrolysate Cc3 and Pa3 supplemented with 20, 40, or 92 g/L glucose.

**Glucose concentration (g/L)**	**Pre-hydrolysate**	**Highest glycerol concentration (g/L)**		**Highest ethanol concentration (g/L)**		***Y*_E/G_ (g/g)**		***Q* (g/L h^–1^)**		***Q*_max_ (g/L h^–1^)**	
		**Fm17**	**ER**	**Fm17**	**ER**	**Fm17**	**ER**	**Fm17**	**ER**	**Fm17**	**ER**
20	-	0.79 ± 0.03	0.74 ± 0.03	8.17 ± 0.38	8.21 ± 0.38	0.41 (80%)	0.41 (80%)	0.31	0.32	0.40	0.40
20	Cc3	0.83 ± 0.03	0.74 ± 0.04	9.34 ± 0.27	8.26 ± 0.21	0.46 (90%)	0.40 (79%)	0.36	0.41	0.47	0.41
20	Pa3	0.80 ± 0.04	0.72 ± 0.03	9.04 ± 0.28	8.01 ± 0.25	0.45 (88%)	0.40 (78%)	0.45	0.31	0.45	0.39
40	*-*	1.51 ± 0.06	1.64 ± 0.07	16.92 ± 0.77	16.95 ± 0.80	0.42 (82%)	0.42 (82%)	0.38	0.39	0.82	0.84
40	Cc3	1.65 ± 0.07	1.55 ± 0.07	18.29 ± 0.43	17.25 ± 0.40	0.45 (88%)	0.42 (82%)	0.66	0.70	0.90	0.85
92	-	3.27 ± 0.13	3.27 ± 0.15	35.56 ± 1.54	36.34 ± 1.39	0.40 (79%)	0.41 (80%)	0.81	0.83	1.33	1.35
92	Pa3	3.80 ± 0.17	3.63 ± 0.16	39.09 ± 1.42	37.60 ± 1.00	0.45 (88%)	0.43 (85%)	0.86	0.85	1.31	1.29

*The same glucose concentrations were supplemented to YNB as reference broth. The highest glycerol and ethanol levels were reported. All experiments were conducted in triplicate (± SD). *Y*_*E/G*_, ethanol yield per gram of consumed glucose calculated on the highest ethanol production and % of theoretical maximum indicated in brackets; *Q*, volumetric productivity at the highest ethanol production.*

Once exposed to the pre-hydrolysate Cc3 (*C. cardunculus*), glucose consumption was completed within the first 20 h by both strains (Figures 1A,B). The industrial yeast Ethanol Red produced higher biomass levels with the final OD_600_ approaching 4.0. On the contrary, the novel yeast strain Fm17 achieved higher ethanol production (9.34 g/L) which was 1.13-fold that of Ethanol Red (Figures 1A,B and Table 6). As such, ethanol yield and maximum productivity (*Q*_max_) values were greater (Table 6). In particular, the selected *S. cerevisiae* Fm17 exhibited an ethanol yield of 0.46 g/g of consumed glucose, corresponding to almost 90% of the theoretical (0.51 g/g), whereas the industrial benchmark stopped only at 0.40 g/g, which corresponds to 79% of the maximum yield.

The strains produced similar fermenting patterns also in the presence of the pre-treatment liquor Pa3 from *P. australis* (Figures 1C,D and Table 6). Biomass yield, detected as OD_600_-values, was higher in the case of *S. cerevisiae* Ethanol Red (Figures 1C,D); meanwhile, ethanol performances were better for the selected strain Fm17 (Figures 1C,D and Table 6), which produced up to 9.04 instead of 8.01 g/L. The resulting ethanol yields were 0.45 and 0.40 g/g of consumed glucose, corresponding to 88 and 78% of the theoretical for *S. cerevisiae* Fm17 and Ethanol Red, respectively (Table 6).

In the control medium, YNB with 20 g/L of glucose (Figures 1E,F), *S. cerevisiae* Fm17, and Ethanol Red readily consumed all the glucose available and OD_600_ levels were higher than those detected in the presence of both Cc3 and Pa3. Furthermore, Ethanol Red reached OD_600_ levels greater than those of Fm17 with values of almost 5.0 (Figures 1E,F). Noteworthy, ethanol levels produced by both strains were lower than those detected in the pre-hydrolysates. *S. cerevisiae* Fm17 and Ethanol Red yielded 8.17 and 8.21 g/L of ethanol, corresponding to 80 and 81% of the theoretical, respectively (Figures 1C,D). Since pre-hydrolysates have a complex chemical composition, presence of additional carbon sources in the medium containing Cc3 and Pa3 is possible, resulting in greater ethanol productions. Furthermore, a higher amount of ethanol produced in the pre-hydrolysates can also be ascribed to the presence of furfural and HMF. Although these chemical compounds exhibit a negative impact on yeast metabolism, their reduction to less toxic compounds can act as a redox sink, thus preventing redox imbalances and increasing final ethanol yield (Wahlbom and Hahn-Hägerdal, 2002; Ask et al., 2013; Favaro et al., 2013a). Furfural and HMF were completely metabolized by the strains (Figures 1A–D). Lower glycerol production observed in Cc3 and Pa3 when compared to the control broth further supports this hypothesis, since glycerol production as redox sink is less favored than furan conversion (Palmqvist et al., 1999; Martín and Jönsson, 2003).

As reported in Table 6, in the presence of both pre-treatment liquors, the volumetric productivities of the yeast strains were generally greater than those recorded in the reference medium (without inhibitor supplementation). This could be due to the presence of weak acids, which can boost the fermentation rate at concentrations below 100 mM (Palmqvist and Hahn-Hägerdal, 2000; Favaro et al., 2013a; Jönsson et al., 2013; Jönsson and Martín, 2016).

**FIGURE 2:**
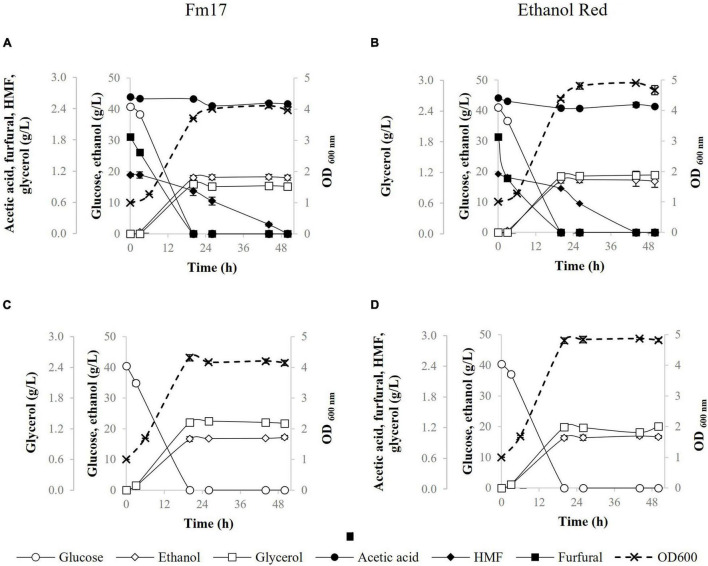
Fermentation performances of *Saccharomyces cerevisiae* strains in YNB broth containing 40 g/L of glucose with or without addition of pre-hydrolysate Cc3 from *Cynara. cardunculus*: Fm17 (**A**: supplemented with Cc3, **C**: reference broth not supplemented with Cc3) and Ethanol Red (**B**: supplemented with Cc3, **D**: reference broth not supplemented with Cc3). The acidity of the medium was adjusted to pH 5.0 with NaOH. The experiment was conducted in triplicate. Error bars correspond to the standard deviation of the means.

**FIGURE 3:**
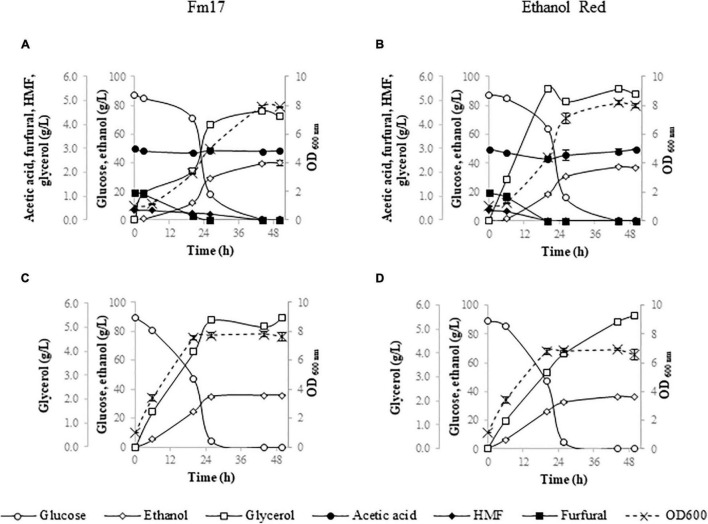
Fermentation performances of *Saccharomyces cerevisiae* strains in YNB broth containing 92 g/L of glucose with or without the addition of pre-hydrolysate Pa3 from *Phragmites australis*: Fm17 (**A**: supplemented with Pa3, **C**: reference broth not supplemented with Pa3) and Ethanol Red (**B**: supplemented with Cc3, **D**: reference broth not supplemented with Pa3). The acidity of the medium was adjusted to pH 5.0 with NaOH. The experiment was conducted in triplicate. Error bars correspond to the standard deviation of the means.

To further assess the fermenting abilities of both strains in industrially relevant conditions, their performances were evaluated in YNB medium formulated with Cc3 (Figure 2) or Pa3 (Figure 3) pre-hydrolysates supplemented up to 40 and 92 g/L of glucose, respectively, to mimic the highest glucose concentration obtained by enzymatic saccharification of each steam-exploded WIS (Cotana et al., 2015a, b; Cavalaglio et al., 2016). pH-value was adjusted to 5.0 with 5 M NaOH. Fermentation medium formulated without Cc3 or Pa3 was used as control (Figures 2C,D, 3C,D).

In the presence of the pre-hydrolysate Cc3 (*C. cardunculus*) and 40 g/L glucose, the strains utilized all glucose available by 20 h of fermentation (Figures 2A,B). Ethanol Red produced higher biomass than Fm17 in both broths (with and without Cc3): final OD_600_ was 4.9 in the control medium and 4.8 in presence of Cc3, amounting to 16% and 20% higher than Fm17, respectively (Figure 2). On the contrary, Fm17 displayed better volumetric productivities and ethanol production (Figure 2 and Table 6). Fm17 and Ethanol Red produced 18.29 g/L and 17.25 g/L of ethanol in the medium formulated with Cc3, respectively, corresponding to 88% and 82% of the theoretical yield (Figures 2A,B and Table 6). Volumetric productivities of Fm17 was significantly higher than those of the industrial yeast, with *Q*_max_ values of 0.90 instead of 0.85 (g/L h^–1^) for *S. cerevisiae* Fm17 and Ethanol Red, respectively (Table 6).

In terms of furans reduction, furfural and HMF were completely metabolized by both strains (Figure 2). In the control medium supplemented with 40 g/L glucose, *S. cerevisiae* Fm17 and Ethanol Red produced lower ethanol levels: 16.92 and 16.95 g/L of ethanol, respectively, corresponding to 82% of the theoretical (Figures 2C,D and Table 6). This finding is consistent with the ethanol performances described earlier (Figure 1), further supporting the hypothesis that the occurrence of additional carbon sources and/or redox sinks in the pre-hydrolysate may have enhanced ethanol production by both strains.

Once exposed to the pre-hydrolysate Pa3 (*P. australis*) with 92 g/L glucose, the strains confirmed their ability to withstand high inhibitor concentrations (Figures 3A,B). However, glucose consumption of both strains took longer than in the reference medium (Figures 3C,D). Nevertheless, ethanol production was high with *S. cerevisiae* Fm17 having again the most promise (39.09 g/L) corresponding to about 88% of the theoretical. The volumetric productivity values of both strains were comparable (Table 6).

As already described and discussed above, ethanol levels in Pa3 were again higher than those detected in the reference broth (YNB supplemented with 92 g/L). *S. cerevisiae* Fm17 and Ethanol Red quickly converted glucose to comparable amounts of alcohol (35.56 and 36.34 g/L, respectively), with an ethanol yield of about 80% of the theoretical (Figures 3C,D and Table 6).

Taken together, the results of small-scale fermentations in the presence of two pre-hydrolysates and increasing concentrations of glucose showed that *S. cerevisiae* Fm17 outcompeted ethanol performances of the reference strain Ethanol Red currently used in industrial bioethanol production (Figures 1–3 and Table 6). Interestingly, the lower glucose concentrations, the greater ethanol yields and productivities (*p* ≤ 0.05) were achieved by the superior yeast *S. cerevisiae* Fm17. Glycerol levels were comparable in both strain fermentations; meanwhile, biomass yields were always higher in the case of the industrial benchmark yeast (Figures 1–3). These findings could be explained considering the ecological origin of Fm17, which has been isolated from grape marcs, an extreme environment with a limited amount of glucose (Favaro et al., 2013a). On the contrary, the industrial strain Ethanol Red has been specifically selected for high alcohol yield and tolerance especially during very high gravity fermentation, typical of the corn ethanol industry where at least 200 g/L glucose is available (Walker and Walker, 2018). As such, the novel *S. cerevisiae* Fm17 seems to be able to withstand better the inhibitors at lower glucose concentrations, reducing biomass yield in favor of ethanol production. These observations are in agreement with the fact that, under anaerobic conditions, yeast cells use alcoholic fermentation of sugars as the sole pathway to obtain energy in the form of ATP for cellular maintenance and, if sufficient ATP is available, for growth. When ATP is utilized for growth, yeast biomass and associated glycerol are produced at the expense of sugars that are not converted to alcohol (Gombert and van Maris, 2015). Furthermore, considering that under SSF or Consolidated BioProcessing settings, glucose levels, released by commercial or, respectively, recombinant enzymes produced by the engineered yeast, do not usually accumulate because of the quick yeast utilization (Hasunuma and Kondo, 2012; Cripwell et al., 2019, 2020), Fm17 should be considered as a very promising lignocellulosic ethanol strain.

In conclusion, this paper was successful in employing undetoxified steam-exploded lignocellulosic residues for second-generation ethanol production. The liquors were used in a close simulation of industrial conditions, considering that as the key point for strain selection.

A cluster of yeast strains demonstrated inhibitor tolerance higher than those of *S. cerevisiae* Ethanol Red, the most used microorganism for lignocellulosic ethanol. This finding is of great value considering that to obtain large additional profits, first-generation ethanol plants strive for an increase of even 1% in ethanol yield. Techno-economical evaluations are in progress to determine the weight of using *S. cerevisiae* Fm17 in the overall process efficiency. Moreover, further studies are on-going to confirm its promising industrial fitness both at higher scale (i.e., bioreactor) and in SSF settings in the presence of WIS collected after steam-explosion of selected lignocellulosic materials.

This study also implies that there are interesting opportunities to isolate or engineer natural yeast variants with performances better than those currently exploited in well-known industrial yeast strains. Moreover, the phenotypic differences between the screened yeast in terms of inhibitor-tolerance indicated that the choice of strain is critical when contemplating the design of a process involving fermentation of lignocellulosic pre-treated materials at industrial scale.

The most burgeoning strain, capable of growing well in undiluted liquors, was also able to ferment in more than one pre-treated feedstocks. This confirmed its great versatility to multiple pre-treated materials, which is one of the requirements for an efficient second-generation ethanol yeast.

## Data Availability Statement

The original contributions presented in the study are included in the article/Supplementary Material, further inquiries can be directed to the corresponding author/s.

## Author Contributions

LC: investigation, data curation, writing original draft, and visualization. NG: data curation, writing original draft, and visualization. MB and SC: commenting revised draft and funding acquisition. LF: conceptualization, methodology, data curation, reviewing original draft, editing, visualization, supervision, and funding acquisition. All authors contributed to the article and approved the submitted version.

## Conflict of Interest

The authors declare that the research was conducted in the absence of any commercial or financial relationships that could be construed as a potential conflict of interest.

## Publisher’s Note

All claims expressed in this article are solely those of the authors and do not necessarily represent those of their affiliated organizations, or those of the publisher, the editors and the reviewers. Any product that may be evaluated in this article, or claim that may be made by its manufacturer, is not guaranteed or endorsed by the publisher.
