# Supplementary material for: Selection of Superior Yeast Strains for the Fermentation of Lignocellulosic Steam-Exploded Residues
Source: Front Microbiol. 2021 Nov 4;12:756032. doi: 10.3389/fmicb.2021.756032 (PMC8601721; doi:10.3389/fmicb.2021.756032)
Supplement: Supplementary file 1 [file Table_1.DOCX]

Supplementary Material

**Supplementary Table 1.** Influence of different lignocellulosic pre-hydrolysates on yeast growth. Yeast strains were cultured in YNB medium containing 20 g/L of glucose and formulated with eight different lignocellulosic pre-hydrolysates. Red and green colors indicate yeast ability or inability to grow in the specific medium, respectively.

|  | ***S. cerevisiae* strains** | | | | | | | |
| --- | --- | --- | --- | --- | --- | --- | --- | --- |
| **Pre-hydrolysate** | **Fm17** | **Fm89** | **Fm90** | **Fm96** | **M2n** | **MEL2** | **YI30** | **Ethanol Red** |
| **Pa1** |  |  |  |  |  |  |  |  |
| **Pa2** |  |  |  |  |  |  |  |  |
| **Pa3** |  |  |  |  |  |  |  |  |
| **Cc1** |  |  |  |  |  |  |  |  |
| **Cc2** |  |  |  |  |  |  |  |  |
| **Cc3** |  |  |  |  |  |  |  |  |
| **Cc4** |  |  |  |  |  |  |  |  |
| **So1** |  |  |  |  |  |  |  |  |
